# Evaluation of Selected Cellulose Macromolecular Properties after Its Chemical Treatment Using Size Exclusion Chromatography

**DOI:** 10.3390/polym15030573

**Published:** 2023-01-22

**Authors:** Tereza Jurczyková, František Kačík, Iveta Čabalová, Kateřina Hájková

**Affiliations:** 1 Department of Wood Processing and Biomaterials, Faculty of Forestry and Wood Sciences, Czech University of Life Sciences Prague, Kamýcka 129, 16000 Prague, Czech Republic; 2 Department of Chemistry and Chemical Technologies, Faculty of Wood Sciences and Technology, Technical University in Zvolen, T. G. Masaryka 24, 96001 Zvolen, Slovakia

**Keywords:** cellulose, wood preservatives, degradation, degree of polymerization, cellulose tricarbanilates, size exclusion chromatography

## Abstract

This work evaluates the effect of using selected inorganic chemicals as the main components of waterborne wood preservative systems on the degradation of the cellulose constituent in wood from model samples. The polymeric properties of cellulose and the homogeneity of the degradation process primarily reflect very well the degree of cellulose deterioration. Whatman papers, as pure cellulose model samples, were impregnated with 10 different 5 wt% solutions of inorganic salts and distilled water and consequently subjected to wet-thermal accelerated aging (T = 85 °C, RH = 65%, for 30 days). The samples were then derivatized to cellulose tricarbanilates (CTCs) through two different procedures (by precipitation in a methanol–water mixture/by evaporation of pyridine from the reaction mixture) and finally analyzed using size exclusion chromatography (SEC). Chemically treated and aged cellulose samples showed different changes in the degree of polymerization (DP) and polydispersity (PD) in terms of untreated non-aged standard caused by different ongoing degradation reactions, such as dehydration, hydrolysis, oxidation, and crosslinking. In general, the lowest degradation rate after treatment by chemicals and after accelerated aging was observed in samples treated by borates, NaCl, and ZnSO_4_·7H_2_O. The greatest depolymerization after treatment and after accelerated aging was caused by sulphates containing NH^4+^, Cu^2+^, and Fe^3+^ cations, with aging by NH_4_Cl and (NH_4_)_2_HPO_4_-treated samples also leading to significant depolymerization. The higher DP values are linked to the precipitated method of CTC preparation, though not for chlorides and phosphates. PD is also generally higher in precipitated and aged samples and is heavily influenced by the presence of low molecular weight products. This paper brings new insights regarding the complex evaluation of the polymeric properties of degraded cellulose by considering all important factors affecting the sample and the analysis itself through the use of statistics. From the statistical point of view, the influences of all factors (solution, aging, method) and their interactions (except aging*method) on DP are statistically significant. The influence of the sample processing method used for analysis of the desired results becomes important mainly in practice. This work recommends the evaporation method for more accurate description of more degraded cellulose.

## 1. Introduction

Although wood is one of the most widespread and essential natural materials, it also has disadvantages. Its most well-known disadvantage is its natural flammability and high ability to generate smoke. For this reason, wood is treated with protective substances. Inorganic salts (borates, phosphates, sulphates, chlorides) are active components of waterborne wood preservatives. These inorganic chemicals induce changes in the molecular structure of wood polymers depending on their concentration, pH value, exposure time, dissociation constant, and other conditions [1,2,3]. Meanwhile, the effect of these compounds when used for wood protection has mostly been observed only in terms of the application process, fixation, leaching, hygroscopicity, corrosivity to metal fasteners, their thermal stability, and dimensional changes in wood mass caused by crystallizing salts from preservative solutions after drying [4,5,6,7,8]. Most researchers have focused on the effect of biocides and flame retardants on the strength properties of the wood under study [1,9,10,11]. Surprisingly little attention has been given to the chemical aspects of this problem, and few controlled experimental studies have been made on the subject [12,13,14].

This work focuses on the evaluation of changes in the macromolecular structure of chemically treated cellulose (a main wood component) with solutions of selected inorganic salts in terms of long-term exposure. However, cellulose isolated from wood always shows a certain degree of degradation caused by agents used in the isolation process, and pure cellulose model samples in the form of Whatman paper were used for better qualitative and quantitative description of changes in the investigated average degree of polymerization (DP) [12,15,16,17].

The extent of cellulose deterioration, i.e., the magnitude of changes in its structure and properties, depends on the extent of damage to its chemical, microscopic, and macroscopic structure. The cellulose synthesized in nature has a certain degree of polydispersity which influences its physical properties. Viscosity and/or the molecular weight distribution (MWD) of cellulose are important parameters for all of its end uses. Therefore, based on determination of MWD and DP, it is possible to accurately evaluate the degradation of cellulose and predict its physical and mechanical properties and ongoing chemical reactions. The result of cellulose degradation is an increased proportion of low molecular weight fractions and a decrease in average DP and polydispersity (PD) [18,19,20,21]. Moreover, DP, as well as crystallinity and crystallite size, have an influence on the thermal stability of cellulose [22,23].

The molecular weight of macromolecules such as cellulose can be determined by absolute and relative methods. Therefore, size exclusion chromatography (SEC) has gained wide acceptance as a preferred method. This method allows for the characterization of MWD and the determination of more of the parameters of the molecular weights. SEC also provides information on degradation fractions and thus helps clarify the mechanism of degradation in cellulose [24,25].

### Formulation of Individual Scientific Goals

To evaluate the influence of the individual inorganic salts, which are active components of fire retardants and biocides, on the degradation of cellulose through the determination of DP via the SEC method.

To compare the DP of cellulose samples degraded by various chemicals among themselves, both with the reference sample (treated only by distilled water) and the standard sample (untreated Whatman paper).

To compare the changes in DP within each series of samples before and after aging.

To assess the changes in DP for each sample series caused by two different methods of CTC sample preparation (evaporation/precipitation in methanol–water mixture) and accordingly identify the samples of treated cellulose with the largest proportion of low molecular weight fractions due to degradation.

To determine whether the solution, aging, method, or combined effect of their interaction (solution*aging, solution*method, method*aging, solution*aging*method) statistically significantly affects the value of the calculated DP.

## 2. Materials and Methods

### 2.1. Material

Whatman filtration papers (Grade No. 1, α-cellulose content >98 wt%, basis weight 87 g/m^2^, air flow rate 10.5 s/100 mL/in^2^) with dimensions of 15 × 100 mm were firstly conditioned to a constant weight at a temperature of 23 ± 1 °C and with a relative air humidity of 50 ± 2% according to the ISO 187 standard [26]. For the treatment of 10 sample series, aqueous solutions of selected chemicals at a 5 wt% concentration level were used, i.e., Na_2_B_4_O_7_·10H_2_O, H_3_BO_3_, CuSO_4_·5H_2_O, ZnSO_4_·7H_2_O, Fe_2_(SO_4_)_3_, NaCl, NH_4_Cl, (NH_4_)_2_SO_4_, (NH_4_)_2_HPO_4_, and NH_4_H_2_PO_4_. Moreover, the standard non-treated unaged samples and reference unaged and aged samples impregnated by distilled water were also included in this experiment. The impregnation of individual series of samples was performed through immersion in a laboratory beaker with a 5 wt% salt solution or distilled water under atmospheric pressure for 30 min. Some pieces of polyethylene mesh were used as interlayers to avoid sticking the individual samples together. The wet-thermal accelerated aging technique according to ISO 5630-3 [27], i.e., at 80 °C and 65% RH for a duration of 30 days, was chosen for aging of one half of each sample series based on the knowledge of previously published studies and existing standards. (Samples releasing ammonia during accelerated aging were separately subjected to elevated temperature and humidity in a second climatic chamber with the intention of preventing the other samples from being contaminated with ammonia.) Prior to testing, all specimens were re-equilibrated to a constant weight [26].

### 2.2. Methods

#### 2.2.1. Derivatization of Cellulose into the Form of Cellulose Tricarbanilates (CTCs)

Accurate determination of molar mass distribution for cellulose samples is a challenging task and a universal method for accurate molar mass distribution analysis of cellulose samples still does not exist [28].

In our work, cellulose tricarbanilates (CTCs) were prepared in two different ways. The first was the modified method of precipitation [29,30]. Briefly, the cellulose samples were dried over silica gel for several days. Anhydrous pyridine (8.0 mL), cellulose (50 mg), and phenyl isocyanate (1.0 mL) were sealed in a 50 mL dropping flask. The flasks were immersed in an oil bath at 70 °C for 72 h. At the end of the reaction, methanol (2.0 mL) was added to the mixture to eliminate excess phenyl isocyanate. The yellow solutions were then added drop wise into a rapidly magnetic stirring 7:3 methanol/water mixture (150 mL). The solids were collected via filtration and washed with 7:3 methanol/water mixtures (1 × 50 mL) and water (2 × 50 mL) to a neutral reaction. The CTC was air-dried overnight and subsequently under vacuum at 60 °C to remove any traces of pyridine. The CTC was then redissolved in 10 mL of THF and filtered through a glass filter (Membrane Solutions, Auburn, WA, USA) with a pore size of 0.7 µm before then being analyzed by SEC. 

In the second method, the pyridine was evaporated from the reaction mixture instead of precipitated, thereby producing a sample containing all the non-volatile products of the derivatization procedure [31]. Subsequently, 2.0 mL of the pyridine reaction mixture was transferred to a 25 mL round bottomed flask and the pyridine was evaporated under vacuum at 40 °C. The syrupy liquid which remained was dissolved in approximately 15 mL of acetone. Following this, 2.0 mL aliquots of the acetone solution were transferred to a 10 mL flask and evaporated with a stream of nitrogen. The residual material was dried overnight under vacuum at 60 °C to remove any traces of pyridine and then redissolved in 10 mL of THF and filtered as above. 

#### 2.2.2. Size Exclusion Chromatography (SEC)

The SEC analysis was carried out on an Agilent 1200 HPLC (Agilent Technologies, Santa Clara, CA, USA) system consisting of an autosampler, quaternary pump, degasser, column thermostat, diode array detector (DAD) working at 240 nm, and differential refractive index detector (DRI). The separation was performed at 35 °C with THF at a flow rate of 1 mL min^−1^ on two PLgel 10 μm (7.5 × 300 mm) MIXED B columns proceeded by a PLgel 10 μm (7.5 × 50 mm) GUARD column (Agilent Technologies, Santa Clara, CA, USA). Data acquisition was carried out with Chemstation software (Agilent Technologies, Santa Clara, CA, USA) and calculations were performed with the Clarity GPC module (DataApex, Prague, Czech Republic). The system was calibrated with polystyrene standards with an average MW in the range of 500–6,035,000 (Polymer Laboratories, Shropshire, UK; Tosoh Corporation, Tokyo, Japan). A universal calibration for determination of molecular weights was used with the constants K = 1.095 × 10^−3^ and α = 0.955 [32]. All SEC results represent the mean of two different samples, and each CTC was chromatographed twice (total of four runs for each sample).

#### 2.2.3. Calculations and Statistical Evaluation

**Figure 1:**
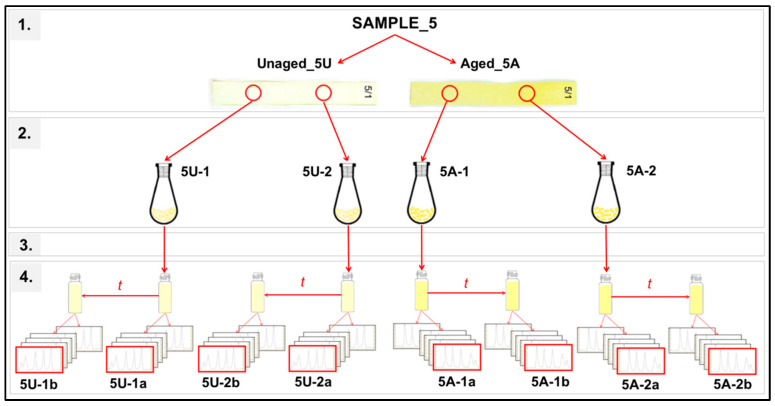
Illustrative overview of three influencing factors (solution, aging, method) on the DP of examined cellulose samples.

The values of molecular weights, the degree of polymerization (DP), and polydispersity indexes (PDI) were calculated after data conversion in Clarity 15.7.1 (DataApex, Praha, Czech Republic). All SEC results represent the mean of two different samples, each CTC was chromatographed twice, and five signals from two detectors (1 × DRI, 4 × DAD) were obtained from each measurement (but only one was used for calculation). The results from chromatographic analysis of CTCs were statistically evaluated by using analysis of variance (ANOVA), and the multiple comparison Duncan’s test was used for the determination of statistically significant differences in DP mean values. The statistical evaluation took 23 situations of variously modified samples into account, as well as the influence of 3 factors (solution, aging, method) and 4 interactions (solution*aging, solution*method, aging*method, solution*aging*method), including 4 repeated measurements of DP for each situation (Figure 1).

The layout of experimental runs and conditions (example for sample 5: treated by Fe_2_(SO_4_)_3_) was as follows: 1. Two pieces were cut from each sample of chemically treated cellulose before and after aging. 2. These pieces of cellulose were converted into the form of CTCs. 3. Two vials with dissolved CTCs in THF were prepared from each CTC sample. 4. One vial was measured twice over time by SEC. This scheme describes the procedure using only one method of CTC preparation. In our case, using two methods, double sampling and measurement were performed for each sample.

## 3. Results and Discussion

Aging and degradation of cellulose due to chemical treatment, elevated temperature, and increased humidity results in a drop in DP and an increase in the proportion of low molecular weight fractions. The adverse consequences of the degradation of cellulose are also reflected in the deterioration of mechanical and optical properties [33,34]. 

### 3.1. Degree of Polymerization (DP)

It has been previously reported that some chemicals tend to reduce cellulose’s molecular weight (MW) and degree of polymerization (DP) [30,35,36,37]. It is apparent from our experiments (Table 1) that there was a greater or a lesser reduction in DP as a consequence of the degradation process caused by presence and action of inorganic salts in the cellulose fiber structure, which was especially evident due to accelerated aging in conditions of elevated temperature and increased relative humidity.

The non-treated unaged standard samples had an average DP of 1444 (when the evaporation method for CTC preparation was used) and 1516 (for the precipitation method). The reference samples, which were only treated with distilled water free of salt cations and anions, recorded a slight increase in DP of about 15.9–15.7% depending on the method of CTC preparation with regard to the standard. This increase in DP, as well as an increase in the crystallinity index caused by preferred degradation of cellulose in its amorphous part, was also observed previously [38,39,40,41]. This anomalous behavior may be also attributed to the formation of covalent intermolecular bonds, i.e., cross-links. Dehydration reactions leading to the formation of predominantly ether bonds have been known to occur during subsequent thermal treatment of cellulose [42]. 

The highest values for DP before accelerated aging, which were higher than the standard samples, were observed in samples treated with NaCl (about 10.3–16.0%) for both methods of CTC preparation. Moreover, in the group of samples where CTCs were prepared via the precipitation method, treatment by Fe_2_(SO_4_)_3_ provided a relatively high DP (about 11.4% higher than standard samples). However, it is evident that this chemical causes a drastic reduction in the DP of cellulose, as it was also determined by the evaporation method. The drop in DP caused by Fe_2_(SO_4_)_3_ was measured at approx. 52.3% in comparison to the evaporated standard, which is a decrease of about 59.2% in comparison to the simultaneously precipitated standard sample. A DP higher than 1000 was observed in celluloses treated by Na_2_B_4_O7·10H_2_O and NH_4_Cl and was confirmed by both methods. That means that these two chemicals should be considered the most gentle salt solutions in the preservation of cellulose, at least in this first impregnation step. The precipitation method provided a DP higher than 1000 for almost all samples besides ammonium phosphates, and the decrease in DP was in the range of 67.7–80.5%. On the other hand, cellulose treated by NH_4_H_2_PO_4_ from precipitated samples provided the lowest DP value from this group, although the group of evaporated samples provided DP values of up to 1000. The highest drop in DP in the group of unaged evaporated samples was observed in celluloses degraded by (NH_4_)_2_SO_4_, CuSO_4_·5H_2_O, and Fe_2_(SO_4_)_3_.

The lowest deterioration in chemically treated cellulose after aging, described by high DP values, was caused by NaCl, Na_2_B_4_O_7_·10H_2_O, and H_3_BO_3_. Even higher DP values than those observed for reference samples were found, with values being in the range of 1.1% for H_3_BO_3_ to 46.9% for NaCl. These findings are confirmed by both the precipitation and evaporation method of CTC preparation. Treatment with ZnSO_4_·7H_2_O also results in higher DP values when the precipitation method is used (even the highest value from aged precipitated samples was about 60.4 % higher than the reference sample). However, there is a slight decrease (about 3.2%) compared to the reference sample when the evaporation method is used. This chemical, as well as borates and NaCl, can be considered as an acceptable chemical for maintaining a high DP even after aging. The most negative influence on cellulose degradation was observed with (NH_4_)_2_SO_4_, (NH_4_)_2_HPO_4_, and Fe_2_(SO_4_)_3_ when the evaporation method was used (the drop in DP was in the range of 95.8–98.6% compared to the evaporated reference sample), and ammonium phosphates and NH_4_Cl demonstrated the greatest negative influence when the precipitation method was used (the drop in DP was in the range of 70.3–87.3% compared to the precipitated reference sample).

### 3.2. Accumulated Degree of Polymerization Loss (ωDP)

Degradation of the cellulose can also be accurately characterized as a decrease in DP, which is calculated by ω_DP_ = 1 − DP_τ_/DP_0_, where ω_DP_ is the accumulated DP loss of cellulose, DP_0_ is the degree of polymerization at an initial time, and DP_τ_ is the real degree of polymerization at the τ time of accelerated aging [43,44]. The same accumulated DP loss values were obtained with both methods of CTC preparation in reference samples. Values corresponding to the samples treated by borates, ZnSO_4_·7H_2_O, and NaCl, i.e., samples with a lower degree of depolymerization, are also in good agreement. On the other hand, a greater than two-fold difference between values obtained from the two different methods of CTC preparation was observed in samples treated by CuSO_4_·5H_2_O and Fe_2_(SO_4_)_3_, where higher values were reported when the evaporation method was used. For samples impregnated by NH_4_Cl and NH_4_H_2_PO_4_, conversely higher values were observed when the precipitation method was used. Based on these results, we can conclude that the lowest difference in DP between treated and aged samples, i.e., the lowest depolymerization caused by accelerated aging in the presence of a certain chemical, can be observed when using ZnSO_4_·7H_2_O and H_3_BO_3_ for treatment. Na_2_B_4_O_7_·10H_2_O and NaCl are, from this point of view, also acceptable. The precipitation method provides the greatest differences in DP between aged and unaged cellulose samples treated by all salts containing ammonia, while the evaporation method confirms that only (NH_4_)_2_SO_4_ and (NH_4_)_2_HPO_4_ from the group of chemicals based on ammonia have a negative effect on treated celluloses in terms of degradation during accelerated aging. 

### 3.3. Polydispersity Index (PDI)

It was reported that the artificial aging of cellulose has an influence on PDI. Emsley et al. [45] found a slight PDI increase in cotton linters aged at 120, 140, and 160 °C. On the other hand, a decrease in PDI was observed in irradiated and chemically or enzymatically treated cellulose [46]. 

Changes in PDI (Table 2) were found for reference samples before aging in comparison to standard samples. The PDI of the reference evaporated sample was about 8.9% lower and the PDI of the reference precipitated sample was up to 20.3% lower than the PDI of the corresponding standard samples. PDI generally differs more in the group of precipitated samples and, of course, after aging. 

In the series of unaged samples, the PDI of most samples remained fairly constant, i.e., values of between 3.15 and 4.04 for the evaporated series and values of between 2.12 and 3.87 for the precipitated series of samples. In these samples, depolymerization proceeds randomly without preferential breakdown of the longest cellulosic chains. When the hydrolysis of the glycosidic bonds is dominant, the ratio increases. Higher PDI values were observed in the group of unaged samples when using ammonium sulphate and phosphates for treatment. The range of PDI values for these samples is from 4.12 to 4.58 for the evaporation method and from 5.36 to 6.52 for the precipitation method. The precipitation method provides slightly higher values than the evaporation method for samples treated by phosphates and all sulphates. On the other hand, the PDI values for the standard and reference samples and borate- and chloride-treated celluloses are lower in the case of precipitation method (up to 40.4% for Na_2_B_4_O_7_·10H_2_O). Fe_2_(SO_4_)_3_ treatment leads to significantly higher PDI values that are approx. 3.3 times higher in the case of the evaporation method and approx. 6.5 times higher in the case of the precipitation method than the corresponding standard samples. This chemical leads, in all cases (independent of method or aging), to an extreme increase in PDI compared to other investigated samples. This means that a great amount of the products of low molecular weight degradation are still present, which thus increases PDI; however, they have no influence on MWD. Additionally, they are usually lost during the precipitation step involving methanol or ethanol when CTCs are prepared [47]. When the precipitation method was used, all three celluloses treated by sulphates containing transition metal cations provided higher PDI values compared to the other samples. 

In the series of aged samples, the calculated PDI values differed quite a lot depending on the method of CTC preparation. The highest PDI values for aged samples obtained via the evaporation method were observed in celluloses treated with NH_4_H_2_PO_4_ (PDI of 31.36, which is also the highest PDI value among all samples) and Fe_2_(SO_4_)_3_ (10.56). High DPI values were also reported for aged precipitated samples impregnated by all sulphates (8.01–11.28). The comparatively higher PDI values observed with precipitation methods were also caused by treatment using phosphates (5.07–6.33). The highest difference in PDI between both methods of CTC preparation was caused when the cellulose was impregnated by ammonium salts (except of NH_4_Cl) and CuSO_4_·5H_2_O. For reference samples, borates, Fe_2_(SO_4_)_3_, NaCl, and NH_4_H_2_PO_4_, the PDI values were lower when the precipitation method was used. 

### 3.4. Statistical Evaluation of the Experiment

The results of determined DP obtained via SEC were statistically evaluated by the three-way analysis of variance (ANOVA) method. 

The final interpretation of ANOVA results with multiple screenings consists of an evaluation of the mutual effect of main factors and the interactions involved in the experiment. To the resulting ANOVA table (Table 3), it should be noted that each mean square is just the sum of squares divided by its degrees of freedom, and the F value is the ratio of the mean squares. Moreover, for determination of the statistically significant difference in mean DP values, the multiple comparison Duncan’s test was used. Statistical significance among mean DP values is demonstrated by a calculated *p*-value lower than α = 0.05.

**Figure 2:**
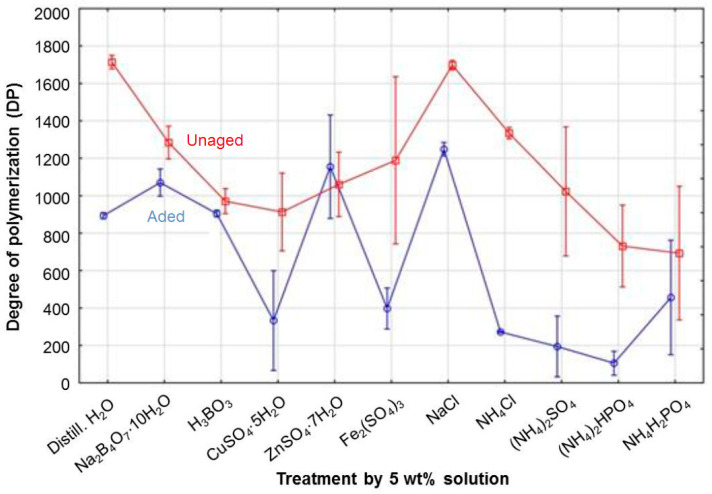
Influence of solution*aging on the degree of polymerization in cellulose.

**Figure 3:**
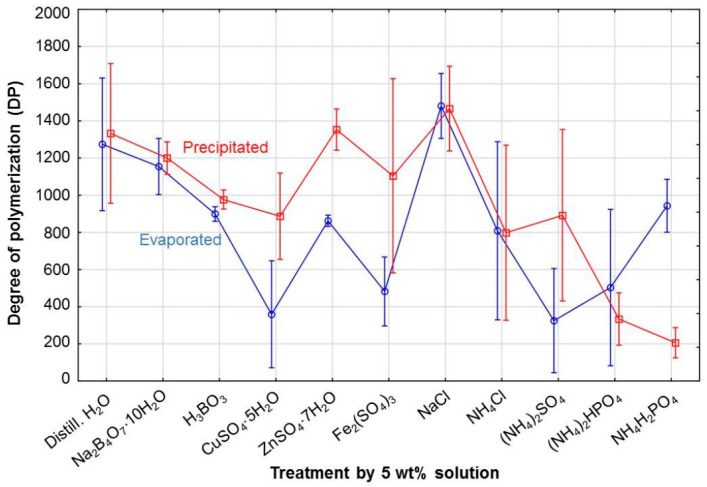
Influence of solution*method on the degree of polymerization in cellulose.

**Figure 4:**
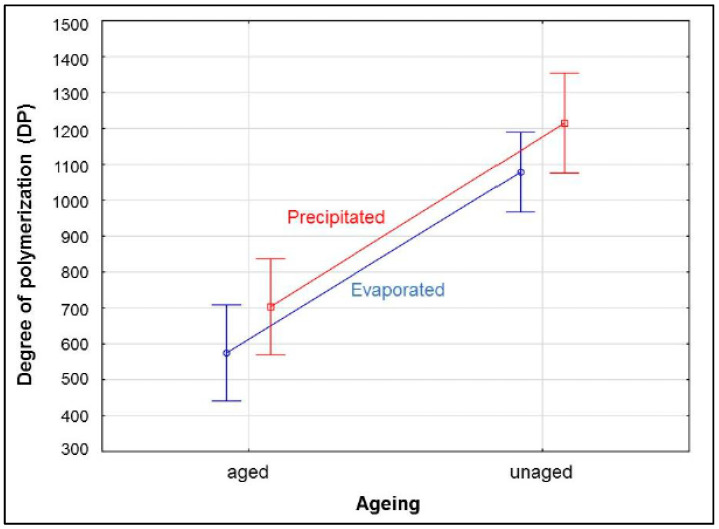
Influence of aging*method on the degree of polymerization in cellulose.

Based on the basic statistical characteristics listed in Table 3, it is possible to suggest that the influences of solution, aging, and method and the interactions of solution*aging, solution*method, and solution*aging*method on DP are statistically significant. Conversely, the influence of aging*method interaction does not provide statistically significant results (*p* > 0.05). From the F-test that tests the differences between two variances, it is apparent that the most important influence on DP from the three factors (solution, aging, method) was aging, while the factor of method was the least significant. From all influencing interactions, the solution*method interaction was the most powerful. The aging*method interaction did not provide statistically significant results because the null hypothesis, in which these two variances are equal at the 0.05 significance level, was rejected. Graphs of cellulose DP showing the influence of various interactions within the three-way ANOVA are shown in Figure 2, Figure 3 and Figure 4.

In general, the precipitation method from the perspective of solution*method interaction provides a higher DP value than the evaporation method (Figure 3). However, when the cellulose was treated by chlorides, the DP values obtained from the evaporation method were slightly higher, and even higher values were observed after treatment with phosphates. Reference samples and samples treated by NaCl, Na_2_B_4_O_7_·10H_2_O, and ZnSO_4_·7H_2_O (only when CTCs were prepared by precipitation in the case of ZnSO_4_·7H_2_O) showed the highest DP values. Low DP values depended more on the method used: (NH_4_)_2_SO_4_, CuSO_4_·5H_2_O, and Fe_2_(SO_4_)_3_ caused significant depolymerization when the evaporation method was used, and NH_4_H_2_PO_4_, (NH_4_)_2_HPO_4_, and NH_4_Cl had a negative effect on DP when the precipitation method was used. Very similar DP values that fell within the tolerance of both performed methods of CTC preparation were provided by samples impregnated by H_2_O (i.e., the reference sample), Na_2_B_4_O_7_·10H_2_O, NaCl, and NH_4_Cl. Similar DP values were also observed in cellulose treated by H_3_BO_3_ and (NH_4_)_2_HPO_4_. This means that MWD is quite uniform without extremely low molecular weight fractions. These low molecular fractions are probably broadly present in samples with sulphate content (NH_4_^+^, Cu^2+^, Zn^2+^, Fe^3+^), as shown by the great difference between DP values for both methods. The evaporation method provided lower DP values due to the presence of low molecular weight fractions that are more reflected by this method. A very similar difference between DP values for the two methods was also observed in the sample treated with NH_4_H_2_PO_4_. However, in this case, a higher DP value is reported for the evaporation method.

The DP values for samples precipitated in the methanol–water mixture presented a very similar statistical trend to the DP values of evaporated samples (Figure 4), although the evaporated samples generally provided lower DP values. For aged evaporated samples, the average DP value for all samples was approx. 17% lower than the corresponding precipitated samples, while values for unaged evaporated samples were approx. 11% lower. This fact indicates that precipitation of the CTC samples in a methanol–water mixture still results in a partial loss of low molecular weight fractions.

The MWD of precipitated CTC samples may not be representative of the MWD of the original cellulose if the cellulose contains a low molecular weight fraction [31]. This is the reason why evaporated CTC samples were also prepared, as they contain (according to assumptions) all the nonvolatile products from a derivatization procedure. Thus, using the evaporation method seems to be suitable for more accurate description of more degraded cellulose treated with various sulphates (differences between the evaporated and precipitated samples are shown in Figure 4), and it may also be useful in general for aged samples with a higher amount of low molecular weight fractions. Similarly, Pitkänen and Sixta [28] showed that size exclusion chromatography of non-derivatized cellulose coupled with multi-angle light scattering (MALS) and differential refractive index (DRI) detectors suffers from low sensitivity in the low molar mass range. They suggest using a combination of two calibration strategies: MALS/DRI for the polymeric region of the cellulose sample and conventional calibration for the oligomeric region.

Comparison of the polymeric properties of treated or aged samples in certain experimental series should always be conducted within one of these methods. For this study, which was focused on cellulose degradation with different chemicals in conditions of wet-thermal accelerated aging, application of the evaporation method is considered to be more accurate. Another reason for using this method, apart from preserving the products of low molecular weight degradation, is the fact that the observed trends in DP decline in the series of evaporated samples correspond more, compared to the precipitation method, with other investigated trends among individual samples within the scope of our previous research works on this topic, e.g., a decrease in cellulose yields, a drop in the degree of cellulose crystallinity [12], the generation of new chromophoric structures, etc. [48].

**Figure 5:**
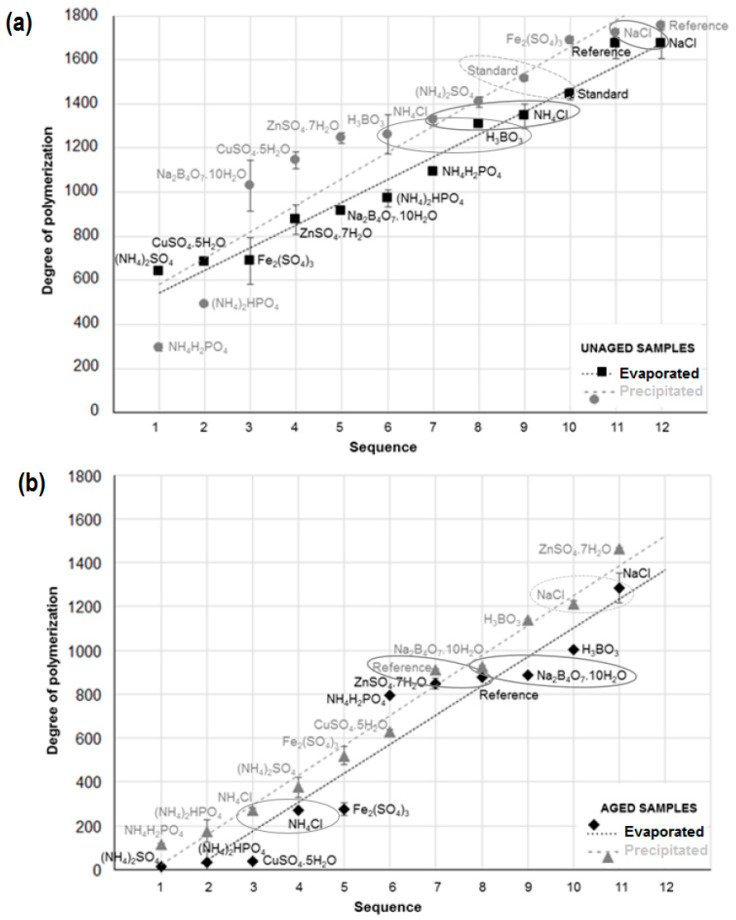
Graphical comparison of average degrees of polymerization for chemically treated cellulose when determination was performed using precipitated and evaporated samples within the group of (**a**) unaged samples and (**b**) samples after accelerated aging.

The results in Figure 5 show that different methods of sample preparation for SEC analysis provide different absolute mean DP values. However, some of the main effects of individual factors on DP have already been found using analysis of variance, though which of these factors have the greatest influence it is still unknown. Therefore, it was necessary to conduct post hoc comparisons between pairs of treatments.

Duncan’s multiple range test is based on the comparison between the range of a subset of sample means and a confidence level mean with a calculated least significant range. This least significant range increases with the number of sample means in the subset. If the range of the subset exceeds the least significant range, then the population means can be considered to represent differences with statistical significance (*p* < 0.01) [49]. If 0.01 < *p* < 0.05, then the parameter demonstrates a moderately statistically significant difference, which is this study can be seen between DP means for unaged precipitated and evaporated standard samples and also between aged NaCl-treated precipitated and evaporated samples. There are also defined pairs of samples between which a statistically significant difference (*p* > 0.05) does not exist. This similarity is present within individual groups of unaged and aged chemically treated celluloses, and moreover between some samples from unaged and aged groups as well. Pairs of samples (precipitated and evaporated) without any significantly different DP values are the H_3_BO_3_-treated, NaCl-treated, and NH_4_Cl-treated celluloses in the unaged group and the reference samples, Na_2_B_4_O_7_·10H_2_O-treated samples, and NH_4_Cl-treated celluloses in the aged group. The pairs of samples across unaged and aged groups that do not have any significant differences in DP means are the CuSO_4_·5H_2_O-treated unaged evaporated and aged precipitated samples, the ZnSO_4_·7H_2_O-treated unaged evaporated and aged evaporated samples, and both (precipitated and evaporated) Na_2_B_4_O_7_·10H_2_O-treated aged and unaged samples.

It is also interesting to highlight the similarities between some of the chemically treated celluloses with standard (non-treated unaged) and reference (distilled water-treated unaged and aged) samples that are evident from Duncan’s test. There was no significant difference between evaporated and precipitated standard samples and precipitated aged ZnSO_4_·7H_2_O-treated samples, nor was there a significant difference between evaporated standard and precipitated unaged (NH4)_2_SO_4_-treated samples. Pairs of samples without any significantly different DP values included the following: unaged evaporated reference sample and unaged precipitated Fe_2_(SO_4_)_3_-treated cellulose/unaged precipitated as well as evaporated NaCl-treated cellulose; unaged precipitated reference sample and unaged precipitated NaCl-treated cellulose; aged evaporated reference sample and aged evaporated as well as precipitated Na_2_B_4_O_7_·10H_2_O-treated cellulose/unaged evaporated Na_2_B_4_O_7_·10H_2_O-treated cellulose/unaged evaporated ZnSO_4_·7H_2_O-treated cellulose. Aged precipitated reference samples had the same relations as aged evaporated reference samples, and there was no significant difference between aged precipitated reference samples and unaged evaporated (NH_4_)_2_HPO_4_-treated cellulose.

## 4. Conclusions

Cellulose degrades due to the effect of inorganic salts, which are active ingredients in biocides and flame retardants. The rate of degradation increases with time and exposure to elevated temperature and humidity. DP, as well as PDI, are reduced during the degradation of cellulose.

Our results show that depolymerization of cellulose was present to a greater or lesser extent in all samples treated with various chemicals, and was also present in the sample treated with distilled water. Acid hydrolysis (catalyzed by metal cations) of glycosidic bonds, oxidation of glucopyranose rings, dehydration, and crosslinking occurred in cellulose degraded by most of these chemicals.

The highest DP values after impregnation were observed in samples treated with distilled water, NaCl, NH_4_Cl, and Na_2_B_4_O_7_·10H_2_O. After aging, the highest DP values were again observed in samples treated by NaCl, Na_2_B_4_O_7_·10H_2_O, and H_3_BO_3_. The greatest depolymerization after treatment and accelerated aging was observed in sulphates containing NH_4_^+^, Cu^2+^, and Fe^3+^ cations, as well as in groups of aged NH_4_Cl and (NH_4_)_2_HPO_4_-treated samples. The lowest accumulated DP loss throughout the process (from initial chemical treatment to the aged form after 30 days) was mainly connected with ZnSO_4_·7H_2_O and borates, and the biggest decline was associated with ammonium salts, especially NH_4_Cl. PD was generally higher in evaporated and aged samples and was heavily influenced by the presence of low molecular weight products. Chemicals such as sulphates (mainly Fe_2_(SO_4_)_3_) and ammonium phosphates (mainly NH_4_H_2_PO_4_) lead, in all cases, to extreme increases in PD compared to other samples.

Therefore, wood preservatives containing ammonia that is bound mainly to phosphates or sulphates and chlorides, as well as wood preservatives containing copper and iron sulphates, are not recommended for the long-term protection of timber due to the large-scale degradation of cellulose constituents. The most acceptable wood protective agents among those studied, in terms of preventing cellulose in wood from being damaged by depolymerization, are borates (primarily alkaline solution of Na_2_B_4_O_7_·10H_2_O), NaCl, and ZnSO_4_·7H_2_O. These assumptions were already confirmed by our previous studies [12,48] dealing with the effect of inorganic salts on wood.

Statistical evaluation of the experiment indicates that aging had the most important influence on DP from the three factors (solution, aging, method), with the factor of method being the least significant.

This study evaluated the appropriateness of using the evaporation method for more accurate description of cellulose samples degraded by sulphates or aging. Duncan’s multiple-range test helped to define which pairs of samples did not have a statistically significant difference. These findings again confirm the close similarity between standard samples (or more precisely the reference sample in this case) and celluloses treated with ZnSO_4_·7H_2_O, Na_2_B_4_O_7_·10H_2_O, and NaCl, which are characterized by a low degree of deterioration.

In any case, SEC analysis proved to be suitable and highly recommended for analysis of chemically treated and aged celluloses. This measurement technique provides important information concerning degraded fractions, thus leading to insights into degradation mechanisms.

## Figures and Tables

**Table 1:** The degree of polymerization (DP) data (mean ± SD) and the accumulated DP loss in chemically treated and aged cellulose samples.

Samples	DP of Treated Samples		DP of Treated and Aged Samples		Accumulated DP Loss (ω_DP_)	
	Evaporated	Precipitated	Evaporated	Precipitated	Evaporated	Precipitated
Standard	1444 ± 3	1516 ± 17	-----	-----	-----	-----
Reference	1673 ± 6	1754 ± 5	875 ± 4	912 ± 4	0.48	0.48
Na_2_B_4_O_7_.10H_2_O	1308 ± 106	1261 ± 114	1002 ± 53	1139 ± 49	0.23	0.10
H_3_BO_3_	913 ± 68	1029 ± 37	885 ± 1	925 ± 9	0.03	0.10
CuSO_4_.5H_2_O	681 ± 5	1145 ± 25	37 ± 1	630 ± 47	0.95	0.45
ZnSO_4_.7H_2_O	877 ± 39	1244 ± 88	847 ± 30	1463 ± 40	0.03	−0.18
Fe_2_(SO_4_)_3_	689 ± 5	1689 ± 21	275 ± 8	520 ± 9	0.60	0.31
NaCl	1675 ± 7	1721 ± 26	1285 ± 23	1212 ± 4	0.23	0.30
NH_4_Cl	1344 ± 53	1326 ± 12	273 ± 1	271 ± 5	0.20	0.80
(NH_4_)_2_SO_4_	638 ± 26	1409 ± 16	12 ± 0	376 ± 3	0.98	0.73
(NH_4_)_2_HPO_4_	972 ± 67	490 ± 20	34 ± 3	176 ± 13	0.97	0.64
NH_4_H_2_PO_4_	1090 ± 71	296 ± 19	796 ± 67	116 ± 6	0.27	0.61

**Table 2:** Polydispersity index (PDI = Mw/Mn) for chemically treated and aged cellulose samples (mean ± SD).

Samples	PDI of Treated Samples		PDI of Treated and Aged Samples	
	Evaporated	Precipitated	Evaporated	Precipitated
Standard	3.71 ± 0.07	2.66 ± 0.03	-----	-----
Reference	3.38 ± 0.03	2.12 ± 0.01	2.94 ± 0.05	2.43 ± 0.03
Na_2_B_4_O_7_.10H_2_O	3.81 ± 0.27	2.27 ± 0.18	3.38 ± 0.16	2.84 ± 0.08
H_3_BO_3_	3.74 ± 0.21	3.15 ± 0.21	3.02 ± 0.01	2.72 ± 0.06
CuSO_4_.5H_2_O	3.69 ± 0.07	8.45 ± 0.26	2.14 ± 0.07	8.53 ± 0.29
ZnSO_4_.7H_2_O	4.04 ± 0.03	11.00 ± 0.70	3.22 ± 0.09	5.83 ± 0.06
Fe_2_(SO_4_)_3_	12.32 ± 0.44	17.18 ± 0.23	10.56 ± 0.67	8.01 ± 0.03
NaCl	3.31 ± 0.10	2.37 ± 0.02	5.45 ± 0.28	3.48 ± 0.05
NH_4_Cl	3.24 ± 0.10	2.98 ± 0.04	2.42 ± 0.01	2.53 ± 0.02
(NH_4_)_2_SO_4_	4.42 ± 0.09	5.73 ± 0.12	1.34 ± 0.03	11.28 ± 0.44
(NH_4_)_2_HPO_4_	4.12 ± 0.03	6.52 ± 0.12	2.24 ± 0.14	6.33 ± 0.41
NH_4_H_2_PO_4_	4.58 ± 0.19	5.36 ± 0.28	31.36 ± 1.78	5.07 ± 0.20

**Table 3:** ANOVA table for three-way analysis of the average degree of polymerization (DP) for the examined cellulose samples influenced by three factors (solution, aging, method) at 15 levels (11 different salt solutions, two types of aging, i.e., unaged and aged samples, two methods of CTC preparation) and their four combined interactions.

Source of Variation	Sum of Squares	Degrees of Freedom	Mean Square (Variance)	F-Test	*p*-Significance Level
Total mean	140,282,339	1	140,282,339	86,771.49	0.000
Solution (a)	18,125,070	10	1,812,507	1121.12	0.000
Aging (b)	11,334,045	1	11,334,045	7010.66	0.000
Method (c)	775,295	1	775,295	479.56	0.000
Solution*Aging (a*b)	5,319,434	10	531,943	329.03	0.000
Solution *Method (a*c)	6,479,499	10	647,950	400.79	0.000
Aging*Method (b*c)	663	1	663	0.41	0.523
Solution*Aging*Method (a*b*c)	1,272,813	10	127,281	78.73	0.000
Random effect	213,403	132	1617	-----	-----

## Data Availability

The data presented in this study are available on request from the corresponding author.
